# Novel Biomarkers Detected by Proteomics Predict Death and Cardiovascular Events in Hemodialysis Patients

**DOI:** 10.3390/biomedicines10040740

**Published:** 2022-03-22

**Authors:** Ping-Hsun Wu, Rie Io Glerup, My Hanna Sofia Svensson, Niclas Eriksson, Jeppe Hagstrup Christensen, Philip de Laval, Inga Soveri, Magnus Westerlund, Torbjörn Linde, Östen Ljunggren, Bengt Fellström

**Affiliations:** 1 Department of Medical Sciences, Uppsala University, 75236 Uppsala, Sweden; 970392@kmuh.org.tw (P.-H.W.); philip.de.laval@medsci.uu.se (P.d.L.); inga.soveri@medsci.uu.se (I.S.); magnus.westerlund@medsci.uu.se (M.W.); torbjorn.linde@medsci.uu.se (T.L.); osten.ljunggren@medsci.uu.se (Ö.L.); 2 Division of Nephrology, Department of Internal Medicine, Kaohsiung Medical University Hospital, Kaohsiung 80708, Taiwan; 3 Center for Big Data Research, Kaohsiung Medical University, Kaohsiung 80708, Taiwan; 4 Department of Nephrology, Aalborg University Hospital, 9000 Aalborg, Denmark; rig@rn.dk (R.I.G.); jeppe.hagstrup.christensen@rn.dk (J.H.C.); 5 Division of Medicine, Department of Nephrology, Akershus University Hospital, 1478 Oslo, Norway; m.h.s.svensson@medisin.uio.no; 6 Uppsala Clinical Research Center, Uppsala University, 75185 Uppsala, Sweden; niclas.eriksson@ucr.uu.se

**Keywords:** death, cardiovascular disease, proteomics, hemodialysis, biomarkers

## Abstract

End-stage kidney disease increases mortality and the risk of cardiovascular (CV) disease. It is crucial to explore novel biomarkers to predict CV disease in the complex setting of patients receiving hemodialysis (HD). This study investigated the association between 92 targeted proteins with all-cause death, CV death, and composite vascular events (CVEs) in HD patients. From December 2010 to March 2011, 331 HD patients were included and followed prospectively for 5 years. Serum was analyzed for 92 CV-related proteins using Proseek Multiplex Cardiovascular I panel, a high-sensitivity assay based on proximity extension assay (PEA) technology. The association between biomarkers and all-cause death, CV death, and CVEs was evaluated using Cox-regression analyses. Of the PEA-based proteins, we identified 20 proteins associated with risk of all-cause death, 7 proteins associated with risk of CV death, and 17 proteins associated with risk of CVEs, independent of established risk factors. Interleukin-8 (IL-8), T-cell immunoglobulin and mucin domain 1 (TIM-1), and C-C motif chemokine 20 (CCL20) were associated with increased risk of all-cause death, CV death, and CVE in multivariable-adjusted models. Stem cell factor (SCF) and Galanin peptides (GAL) were associated with both decreased risk of all-cause death and CV death. In conclusion, IL-8, TIM-1, and CCL20 predicted death and CV outcomes in HD patients. Novel findings were that SCF and GAL were associated with a lower risk of all-cause death and CV death. The SCF warrants further study with regard to its possible biological effect in HD patients.

## 1. Introduction

Cardiovascular (CV) disease is the major cause of death in patients with chronic kidney disease (CKD) [1]. Patients with CKD have a high burden of traditional risk factors (e.g., diabetes mellitus, hypertension, smoking, and dyslipidemia) and non-traditional risk factors (e.g., endothelial dysfunction, calcification, volume overload, mineral bone disease, anemia, and inflammation) for CV disease [2]. Biomarkers help classify disease phenotypes and predict complications, both in the general population and in patients with CKD [3]. Apart from several established CV disease risk factors in CKD patients [4], other biomarkers have also been associated with CV diseases, such as C-reactive protein (CRP) [5], interleukin (IL)-6 [6,7], fibroblast growth factor 23 (FGF23) [8,9], brain natriuretic peptide (BNP), N-terminal pro-brain natriuretic peptide (NT-proBNP) [10,11], cardiac Troponin I and Troponin T [5,12,13]. Given the complex etiology and high risk of CV disease in hemodialysis (HD) patients, both searching for new biomarkers and evaluating the predictive performance of composite biomarkers is still of interest.

Previous studies have shown that the measurement of 92 selected CV proteins using a proximity extension assay (PEA) could predict atherosclerosis in the general population [14] as well as myocardial infarction and stroke in patients with diabetes [15]. So far, few studies have used a proteomics approach to identify biomarkers that may predict CV death in patients with end-stage renal disease [16]. The aim of this study was to evaluate the predictive value of the PEA-based proteomics for all-cause death, CV death, and composite vascular events (CVEs) in a prospective cohort of HD patients with long follow-up.

## 2. Materials and Methods

### 2.1. Subjects, Study Design, and Follow-Up Events

This prospective cohort study with 5 years of follow-up included patients between December 2010 and March 2011. All prevalent patients receiving HD at five different dialysis facilities in Jutland, Denmark, were assessed for eligibility. We enrolled chronic HD patients above 18 years and excluded patients with acute kidney injury or who were unable to understand informed consent. Medical history was obtained from both patient interviews and medical charts. The primary cause of end-stage kidney disease (ESKD) included hypertension, diabetes, glomerulonephritis, polycystic kidney disease, and others. Baseline blood samples were collected immediately before the start of a dialysis session. After centrifugation, serum samples were separated and stored at −80 °C for later analysis. The time interval between subjects’ inclusion and prior dialysis session was recorded. A single physician reviewed medical records for all participants at study entry and follow-up. The recorded events during follow-up included the cause of death and CVEs defined as acute myocardial infarction (AMI), unstable angina, cerebrovascular disease (ischemic stroke or transient ischemic attack), hemorrhagic stroke, or peripheral artery disease (PAD) events. A detailed definition of CVEs is presented in the supplementary materials. All patients were followed for 5 years or until death, loss to follow-up, dialysis modality change, the cessation of dialysis treatment, or kidney transplantation. The study protocol was approved by the Regional Research Ethics Committee of The North Jutland Region (protocol N-20100041), and the study complied with the Declaration of Helsinki.

### 2.2. Clinical Biochemistry and PEA Proteomics Biomarkers Profiling

Regular biochemical measurements were performed using standard methods at the Department of Clinical Biochemistry, Aalborg University Hospital. CRP (measured using Tina-quant^®^ C-Reactive Protein Gen.3) was analyzed at the Department of Clinical Biochemistry, Aalborg University Hospital. Serum samples were assessed with the Proseek Multiplex 96 × 96 proximity extension assay (PE) using the Cardiovascular I panel (Olink Bioscience, Uppsala, Sweden). The assay simultaneously measures 92 proteins (Table S1) with two particular antibodies for each protein linked to a Polymerase Chain Reaction (PCR) reporter sequence. Each sample includes two incubations, one extension, and one detection control to determine the lower detection limit and normalize the measurements using the Fluidigm BioMark HD real-time PCR platform. The reported values are log2-transformed for subsequent analysis. The validation study of the assay, which included 90 proteins and 7 samples analyzed in nine separate runs, found that the mean intra-assay and inter-assay coefficients of variation were 8% and 12%, respectively, and an inter-site variation of 15% was found [17]. The multiplex PEA performed well in complex samples consuming only 1 µL of sample per test. Normalized protein expression (NPX) values are generated from quantitative PCR quantification cycle values. All assay characteristics, including detection limits and measurements of assay performance and validations, are available from the manufacturer’s webpage (https://www.olink.com/resources-support/document-download-center/ [accessed date 21 March 2022]). The values obtained correlate to the concentration of the target protein without giving absolute concentration values [14]. Quality control of OLINK proteomics data was carried out using their standard quality control pipeline (QC). Two proteins (IL-4 and Melusin) were excluded because of missing data or the low quality of proteomics.

### 2.3. Statistical Analyses

Baseline characteristics are presented as the mean ± standard deviations (SD) or the median (interquartile range) for continuous variables and percentages for categorical variables. The relationship between clinical factors and PEA-based proteins was analyzed using Spearman correlation or Point Biserial correlation as appropriate. To examine the risk of clinical outcomes (all-cause death, CV death, and CVEs) in relation to PEA-selected protein biomarkers, univariable and multivariable Cox regression analyses were performed using continuous models (hazard ratios (HRs) per 1 standard deviation (SD) increase in NPX value) for CV biomarkers. Multivariable Cox proportional hazard models were adjusted for age, gender, smoking status, cause of ESKD, dialysis vintage, dialysis treatment time per week, diabetes, previous myocardial infarction, previous unstable angina, previous cerebrovascular disease, previous peripheral artery disease, the blood level of albumin, phosphate, and CRP. Results were reported as hazard ratios (HRs) with 95% confidence intervals (CIs), and a two-tailed *p* < 0.05 was considered statistically significant. All statistical analyses were performed using R software version 4.1.0 (R Foundation for Statistical Computing; https://www.r-project.org) [accessed date 3 January 2022].

## 3. Results

**Figure 1:**
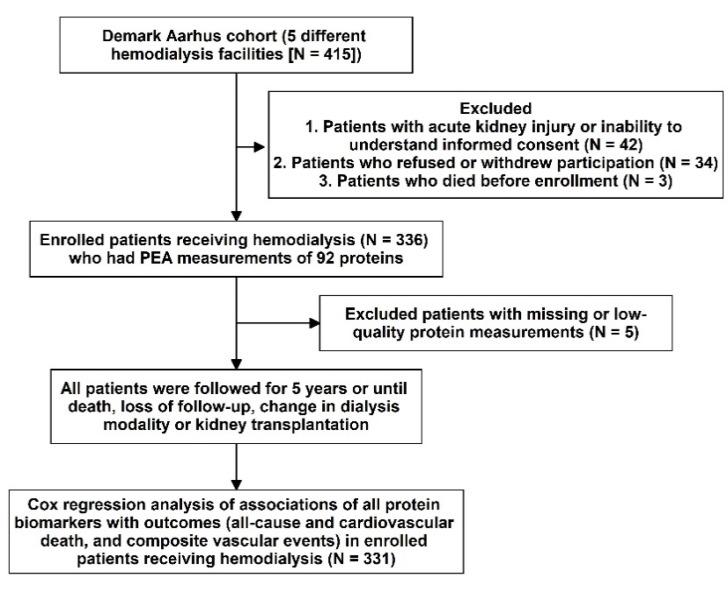
Study design and flowchart.

A total of 336 HD patients met the inclusion criteria. In total, 5 patients with missing or low-quality proteomics protein data were excluded, leaving 331 patients to the final analysis (Figure 1).

### 3.1. Associations between Proteins and Outcome Events

**Figure 2:**
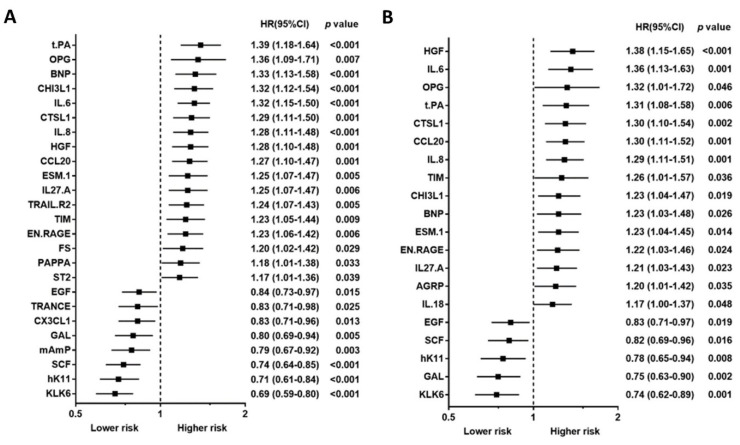
Unadjusted hazard ratio (**A**) and adjusted hazard ratio (**B**) of top proximity extension assay-based protein biomarkers associated with all-cause death in hemodialysis patients. Hazard ratios (HRs) were presented as a per SD increase in NPX value on proteomics. Multivariable Cox regression models were adjusted for age, gender, smoking status, cause of end-stage kidney disease, diabetes, previous myocardial infarction, previous unstable angina, previous cerebrovascular disease, previous treatment for peripheral artery disease, albumin, phosphate, and C-reactive protein, dialysis modality, dialysis treatment time per week, and dialysis vintage.

**Figure 3:**
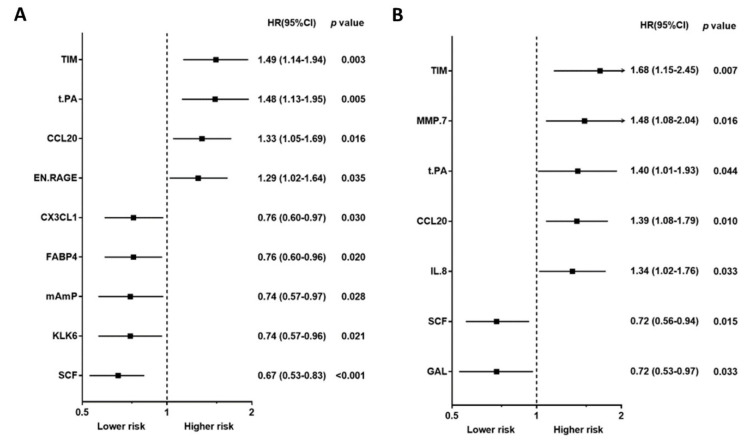
Unadjusted hazard ratio (**A**) and adjusted hazard ratio (**B**) of proximity extension assay-based protein biomarkers associated with cardiovascular death in hemodialysis patients. Hazard ratios (HRs) were presented as a per SD increase in NPX value on proteomics. Multivariable Cox regression models were adjusted for age, gender, smoking status, cause of end-stage kidney disease, diabetes, previous myocardial infarction, previous unstable angina, previous cerebrovascular disease, previous treatment for peripheral artery disease, albumin, phosphate, and C-reactive protein, dialysis modality, dialysis treatment time per week, and dialysis vintage.

**Figure 4:**
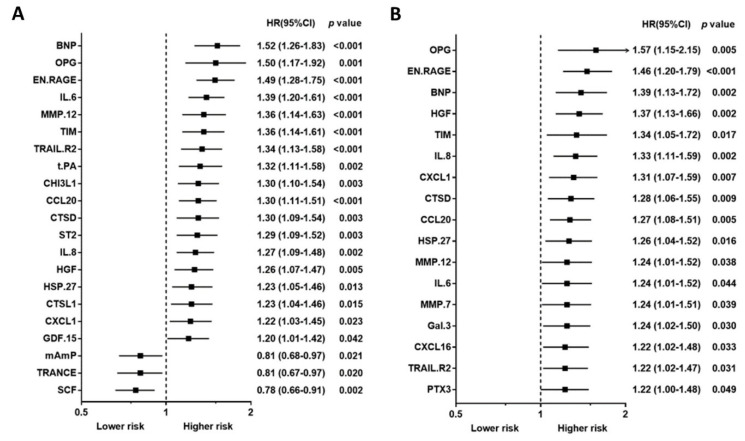
Unadjusted hazard ratio (**A**) and adjusted hazard ratio (**B**) of proximity extension assay-based protein biomarkers associated with a composite vascular event in hemodialysis patients. Hazard ratios (HRs) were presented as a per SD increase in NPX value on proteomics. Multivariable Cox regression models were adjusted for age, gender, smoking status, cause of end-stage kidney disease, diabetes, previous myocardial infarction, previous unstable angina, previous cerebrovascular disease, previous treatment for peripheral artery disease, albumin, phosphate, and C-reactive protein, dialysis modality, dialysis treatment time per week, and dialysis vintage.

Baseline characteristics, including traditional biomarkers, are presented in Table 1 for study, subject-categorized according to the event type. The 92 PEA-based proteomics biomarkers in the entire cohort are shown in Table S1. During the follow-up period, 167 patients (50.4%) died, of which, 62 deaths (18.7%) were classified as CV deaths, and 140 patients (42.3%) experienced a CVE. The mean follow-up times for death and non-death were 797 ± 520 days and 1187 ± 710 days, separately. The outcome (all-cause death, cardiovascular death, and composite vascular events) association in PEA-based proteins in the Cox regression model with multiple testing via false discovery rate (FDR) are presented in Table S2. The associations between single PEA-based proteins and outcomes (all-cause death, CV death, and CVEs) are shown in Figure 2, Figure 3 and Figure 4. In multivariable Cox models, adjusted for confounders, there were 15 proteins associated with increased risk of all-cause death (Figure 2B), 5 proteins associated with increased risk of CV death (Figure 3B), and 17 proteins associated with increased risk of CVEs (Figure 4B). In addition, the adjusted Cox models also found five proteins related to a decreased risk of all-cause death (Figure 2B) and two proteins related to a decreased risk of CV death (Figure 3B). The detailed associations between all PEA-based proteins and clinical biomarkers and clinical outcomes in HD patients are presented in Figures S1–S3.

**Figure 5:**
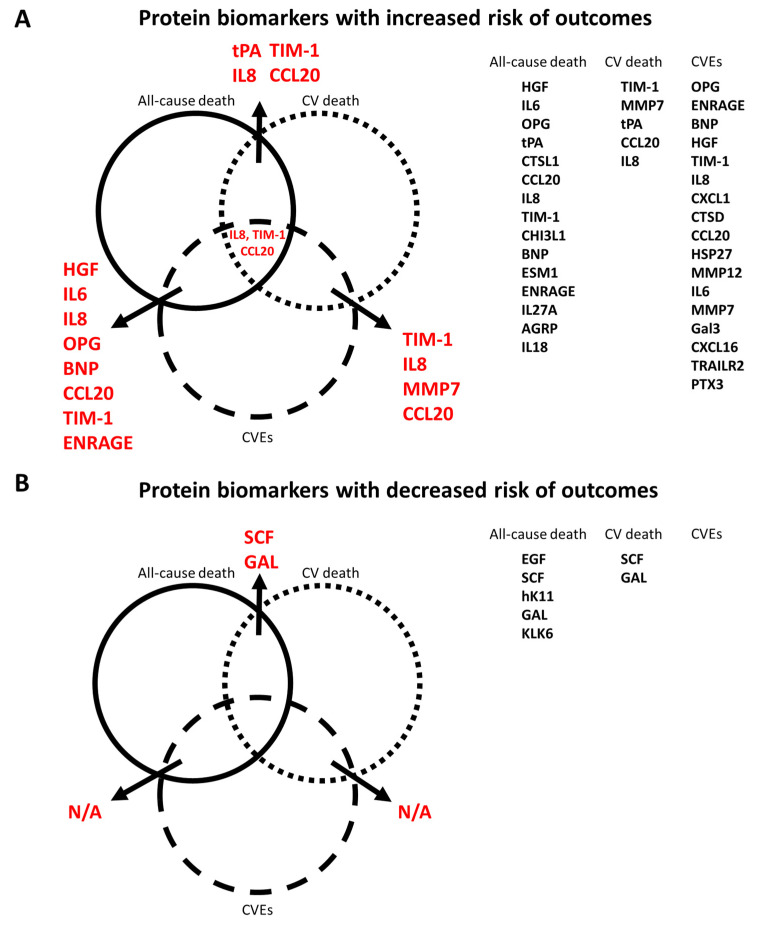
Venn diagram of all PEA-based protein biomarker and outcome (all-cause death, cardiovascular death, and composite vascular events) relationships. (**A**) Protein biomarkers related to increased risk of outcomes. (**B**) Protein biomarkers related to decreased risk of outcomes.

Using a Venn diagram to summarize the PEA-based proteins associated with outcomes in the multivariable-adjusted model, interleukin-8 (IL-8), T-cell immunoglobulin and mucin domain 1 (TIM-1), and C-C motif chemokine 20 (CCL20) were found as biomarkers for all-cause death, CV death, and CVEs (Figure 5). The HRs of IL-8 in the multivariable-adjusted model were 1.29 (95% CI 1.11–1.51, *p* = 0.001) for all-cause death, 1.34 (95% CI 1.02–1.76, *p* = 0.036) for CV death, and 1.33 (95% CI 1.11–1.59, *p* = 0.002) for CVEs (Figure 2B, Figure 3B and Figure 4B). The HRs of TIM-1 in the multivariable-adjusted model were 1.26 (95% CI 1.01–1.57, *p* = 0.036) for all-cause death, 1.68 (95% CI 1.15–2.45, *p* = 0.007) for CV death, and 1.34 (95% CI 1.05–1.72, *p* = 0.017) for CVEs (Figure 2B, Figure 3B and Figure 4B). The HRs of CCL20 in the multivariable-adjusted model were 1.30 (95% CI 1.11–1.52, *p* = 0.001) for all-cause death, 1.39 (95% CI 1.08–1.79, *p* = 0.010) for CV death, and 1.27 (95% CI 1.08–1.51, *p* = 0.005) for CVEs (Figure 2B, Figure 3B and Figure 4B). Interestingly, stem cell factor (SCF) and Galanin peptides (GAL) were associated with reduced risk of both all-cause death and CV death (Figure 5B). The HRs of SCF in the multivariable-adjusted models were 0.82 (95% CI 0.69–0.96, *p* = 0.016) for all-cause death and 0.72 (95% CI 0.56–0.94, *p* = 0.015) for CV death. The HRs of GAL were 0.75 (95% CI 0.63–0.90, *p* = 0.002) for all-cause death and 0.72 (95% CI 0.53–0.97, *p* = 0.033) for CV death (Figure 2B and Figure 3B).

### 3.2. Relationship between Clinical Factors and PEA-Based Proteins

The correlation between baseline characteristics and PEA-based proteins is shown in Figure S4. In particular, IL-8 was negatively correlated with phosphate and albumin level. TIM was negatively correlated with the duration of renal replacement therapy, dialysis modality, dialysis treatment time per week, and albumin and was positively correlated with age, residual urinary output, a diagnosis of diabetes or previous myocardial infarction, and statin treatment. Furthermore, CCL20 was found to be negatively correlated with albumin and positively correlated with PAD. SCF was negatively correlated with male gender, residual urinary output, and PAD history and positively correlated with albumin level.

## 4. Discussion

We identified 15 PEA-based proteins associated with increased risk of all-cause death independent of established risk factors. Three of these proteins (IL-8, TIM-1, and CCL20) were also associated with an increased risk of CV death and CVEs. On the contrary, five PEA-based proteins were associated with decreased risk of all-cause death, but only SCF and GAL were related to a decreased risk of CV death. As for CVEs, 17 PEA-based proteins were independently associated with a higher risk of CVEs, but none were related to decreased risk.

### 4.1. Comparison with Other PEA-Based Proteomics Studies in Patients with Kidney Disease

In a recent PEA-based proteomics study in the cohort Mapping of Inflammatory Markers in Chronic Kidney disease (MIMICK), TIM-1, matrix metalloproteinase (MMP)-7, tumor necrosis factor receptor 2 (TNFR2), IL-6, MMP-1, BNP, ST2, hepatocyte growth factor (HGF), TNF-related apoptosis-inducing ligand receptor 2 (TRAIL-R2), spondin-1, and FGF23 showed significant associations with CV death after adjusting for age and sex in 183 HD patients [16]. Among proteins reported in the MIMICK study, several proteins were also correlated with CVEs in our study, such as TIM-1, MMP-7, IL-6, BNP, HGF, and TRAIL-R2. However, some proteins associated with CV death in our study (e.g., tPA, CCL20, GAL, IL-8, and SCF) were not associated with CV death in the MIMICK cohort. Importantly, TIM-1, also known as kidney injury molecule-1 (KIM-1), was the most important risk marker for CV death in the MIMICK cohort [16], a result which was confirmed in the present study (Figure 2B). On the contrary, while our results showed that SCF was associated with a lower risk of CV death, there was only an insignificant lower risk of CV death associated with SCF in the MIMICK cohort [16]. There could be several reasons for the differences observed between the two studies, including the smaller sample size in the MIMICK cohort (n = 183), longer dialysis vintage in our study, and adjustment for different confounders. In addition, the definition of CV death was validated by a physician in our study, while the International Classification of Diseases (ICD) diagnostic codes definition was used in the MIMICK cohort. In addition, in a discovery-validation study of subjects with non-dialysis CKD, MMP-12 was significantly associated with an increased risk of major adverse CV events (fatal or non-fatal myocardial infarction or fatal and non-fatal stroke) [18]. This is in line with our results showing that MMP-12 is an independent biomarker and may predict CVEs in HD patients.

### 4.2. Comparison with Other Biomarker Studies in Patients with Kidney Disease

Chronic inflammation is an essential CV risk factor in patients with kidney disease and is characterized by the enhanced production of CRP and other inflammatory mediators, including IL-6 and IL-8. These cytokines and chemokines have previously been associated with all-cause death and CV death in HD patients [6,7,19,20]. In line with previous studies in HD patients [21], EN-RAGE, another inflammatory biomarker, was also positively associated with CVEs. Furthermore, we found that TRAIL-R2 (a member protein of the TNF-receptor superfamily) and OPG, reflecting the bone–vascular axis, were related to a higher risk of CVEs. OPG has previously been described as a CV marker for all-cause death in HD patients [22,23,24], and TRAIL-R2 is present in human atherosclerotic lesions with higher expression levels in vulnerable plaques than in stable ones [25].

### 4.3. Potential Mechanisms and Clinical Studies of Novel Biomarkers

Several novel biomarkers, such as HGF, CCL20, tPA, GAL, and SCF, were related to death or CV outcomes.

HGF is a pleiotropic cytokine that regulates several biological processes, such as cardiometabolic activities, inflammation, angiogenesis, and tissue repair [26]. HGF is associated with coronary artery disease [27] and ischemic stroke [28] in patients without CKD. In HD patients, higher levels of HGF are associated with concentric left ventricular geometry [29] and cerebral infarction [30]. This may be related to leukocyte activation during HD [31]. Our study is the first to demonstrate that HGF is associated with an increased risk of both all-cause death and CVEs in HD patients.

Previous studies have shown that CCL20 excretion is increased in stage 5 CKD patients compared with healthy subjects and stage 1–3 CKD patients [32]. Furthermore, patients with ischemic heart disease have higher circulating CCL20 [33], and CCL20 is expressed in atherosclerotic plaques [34]. A recent study demonstrated that CCL20 was associated with increased CV events in stage 3–5 CKD patients [18]. CCL20 contributes to vascular endothelial inflammation [35] and triggers pathways similar to those activated by low-density lipoprotein cholesterol [34]. IL-6, IL-8, and CCL20 are cytokines or chemokines related to the Th17 CD4 lymphocyte [36]. Several studies have shown a critical role of Th17 cells in arteriosclerosis development [37]. The genetic deletion of CCL20 receptors in Apoe−/− mice decreases atherogenesis and endothelial inflammation [38]. Therefore, we speculated that the accumulation of CCL20 might significantly impact the risk of CV disease in HD patients.

tPA is a glycoprotein involved in coronary plaque rupture [39] and is considered a marker in regulating endogenous fibrinolysis [40]. In the general population without kidney disease, there is an association between elevated circulating levels of tPA and subsequent coronary heart disease [40]. In general, the circulating levels of tPA are elevated in dialysis patients compared with healthy controls [41], and circulating levels of tPA in HD patients are positively associated with several CV risk factors, such as age, smoking, blood pressure, and CRP level [41].

GAL is an endocrine hormone of the central and peripheral nervous systems involved in central CV regulation that affects heart rate and blood pressure [42]. However, functional properties of the galaninergic system are not fully elucidated for cardiac diseases. In the animal model, GAL can limit myocardial infarction size, improve postischemic cardiac function recovery [43], and suppress myocardial apoptosis and mitochondrial oxidative stress in cardiac hypertrophic remodeling [44]. These basic studies could support our results of the negative association between GAL and CV death in HD patients.

A novel finding was the association between high SCF levels and lower all-cause or CV death. Similar results were described in the general population (Malmö Diet and Cancer study) [45] and stable coronary heart disease patients [46]. SCF is involved in vasculogenesis and cardiac repair by stimulating the recruitment and activation of bone-marrow-derived stem cells and tissue-resident progenitors [47]. An increase in SCF expression occurs naturally in response to myocardial infarction, which mediates the migration of c-kit+ cardiac and bone marrow cells to the injured area for cardiac remodeling [48]. We found that circulating SCF levels were positively correlated with albumin. It is well known per se that albumin is a predictive marker in HD patients. We speculate that SCF may play a protective role in vascular injury and may reflect the clinical nutrition status in HD patients.

Regarding the correlation between clinical variables and PEA-based protein biomarkers, phosphorus level was negatively correlated with IL-8 and tPA. In previous studies, a J-shaped relationship between circulating phosphate level and mortality in HD patients has been demonstrated [49,50]. Higher phosphorus levels may lead to vascular calcification, while lower levels represent a poor nutritional state [49,50]. Therefore, our finding of a negative correlation between phosphorus level and IL-8 may indicate the connection to malnutrition–inflammation status in HD patients.

### 4.4. Strengths and Limitations

The strengths of our investigation include the longitudinal study design and the use of the proteomics chip for multiple simultaneous biomarker measurements. However, some limitations need to be addressed. First, this study focused mainly on Caucasian patients receiving HD therapy in Scandinavia, so extrapolations to non-Caucasian HD patients or CKD patients not yet on dialysis should be made cautiously. Second, the study used single assessments of the proteomic assay, so there could be potential misclassification and short-term variability. Third, the proteins on the chip were pre-selected based on literature reports or experimental studies on CV disease, so it is not an untargeted proteomics approach. However, an untargeted method is more potent and ideal for discovering novel biomarkers [51]. In addition, better validation of candidate protein biomarkers by applying them to an additional different type of protein analysis should be considered to avoid the risk of the high-dose hook effect in a single-method approach. Fourth, the scale of the proteomics assay is not readily convertible to absolute concentrations for comparisons with previous studies, and no relevant cutoff limits exist. Fifth, the number of statistical tests performed in this manuscript increases the risk of spurious findings; hence, novel previously published findings need to be replicated in an independent cohort of patients. In the Cox regression analyses, we assumed a linear effect of the biomarkers, whereas the association might be nonlinear. Considering many covariates differ across levels of biomarkers and affect the risk of disease, the differences in unadjusted and adjusted results could be expected. The variables we adjusted for could confound different biomarker results between unadjusted and adjusted models. This process could give researchers a hint that these biomarkers were affected by clinical factors. Finally, since CV and kidney diseases are tightly linked, our biomarker candidates may not participate directly in CV events. The PEA-based proteomics analysis could be examined in early kidney disease subjects to confirm the biomarker candidates related to CV disease in all CKD stages.

## 5. Conclusions

Using the PEA proteomics biomarkers approach, we identified several novel biomarkers independently associated with all-cause death, CV death, and CVEs in HD patients. Higher IL-8, TIM-1, and CCL20, as well as lower SCF and GAL, were associated with poor outcomes. SCF is novel and warrants validation in future studies and needs to be evaluated for its potential biological impact in HD patients.

## Figures and Tables

**Table 1:** Baseline demographic characteristics of hemodialysis patients with different outcomes.

	All-Cause Death	Cardiovascular Death	Composite Vascular Event	No Event	Significance
Patients (n)	167	62	140	122	
Age, years	68.9 ± 12.5	68.1 ± 11.9	67.5 ± 12.0	58.9 ± 16.3	***, ###, †††
Males, n (%)	115 (69%)	47 (76%)	96 (69%)	74 (61%)	NS, NS, NS
Smoking					NS, NS, †
Current smoker, n (%)	39 (23%)	18 (29%)	43 (31%)	33 (27%)	
Never smoker, n (%)	55 (33%)	17 (27%)	33 (24%)	48 (39%)	
Former smoker, n (%)	73 (44%)	27 (44%)	64 (46%)	41 (34%)	
Cause of end-stage kidney disease					*, ###, †††
Hypertension, n (%)	24 (14%)	13 (21%)	22 (16%)	15 (12%)	
Diabetes mellitus, n (%)	36 (22%)	18 (29%)	39 (28%)	14 (12%)	
Glomerulonephritis, n (%)	16 (10%)	3 (5%)	9 (6%)	21 (17%)	
Cystic disease, n (%)	10 (6%)	2 (3%)	9 (6%)	17 (14%)	
Others, n (%)	81 (49%)	26 (42%)	61 (44%)	55 (45%)	
Hemodialysis vintage, days	1533 ± 1592	1366 ± 1427	1468 ± 1505	1337 ± 1714	NS, NS, NS
Renal replacement therapy vintage, days	2041 ± 2385	1706 ± 1934	2032 ± 2473	2344 ± 2955	NS, NS, NS
Previous kidney transplant, n (%)	8 (5%)	3 (5%)	7 (5%)	11 (9%)	NS, NS, NS
Residual renal function of daily urine amount					NS, NS, NS
<50 mL/day, n (%)	63 (38%)	23 (37%)	53 (38%)	41 (34%)	
50–500 mL/day, n (%)	28 (17%)	9 (15%)	24 (17%)	28 (23%)	
>500 mL/day, n (%)	76 (46%)	30 (48%)	63 (45%)	53 (43%)	
Dialysis modality					NS, NS, NS
Hemodialysis, n (%)	145 (87%)	55 (89%)	116 (83%)	106 (87%)	
Hemodiafiltration, n (%)	22 (13%)	7 (11%)	24 (17%)	16 (13%)	
Dialysis treatment time per week, hours	11.1 ± 2.6	11.4 ± 2.5	11.3 ± 2.7	11.2 ± 2.6	NS, NS, NS
Time interval between subjects’ inclusion and prior dialysis session, hours	59.7 ± 23.0	57.5 ± 23.8	58.9 ± 22.0	57.5 ± 20.7	NS, NS, NS
Comorbidities					
Diabetes mellitus, n (%)	53 (32%)	24 (39%)	53 (38%)	22 (18%)	*, ##, †††
Type 1 diabetes mellitus, n (%)	10 (6%)	6 (10%)	12 (9%)	9 (7%)	
Type 2 diabetes mellitus, n (%)	43 (26%)	18 (29%)	41 (29%)	13 (11%)	
Previous myocardial infarction, n (%)	40 (24%)	17 (27%)	30 (21%)	16 (13%)	*, #, NS
Previous unstable angina, n (%)	54 (32%)	23 (37%)	44 (31%)	20 (16%)	**, ##, ††
Previous cerebrovascular disease, n (%)	38 (23%)	19 (31%)	42 (30%)	16 (13%)	NS, ##, ††
Previous peripheral artery disease, n (%)	43 (26%)	20 (32%)	43 (31%)	15 (12%)	**, ##, †††
Statin treatment, n (%)	61 (37%)	22 (36%)	58 (41%)	37 (30%)	NS, NS, NS
Clinical laboratory data					
Albumin, (g/L)	38.17 ± 4.09	38.56 ± 4.19	38.29 ± 4.02	40.11 ± 3.13	***, #, †††
C-reactive protein (mg/L)	7.56 (16.20)	7.02 (12.72)	7.08 (14.52)	4.46 (7.68)	***, ##, †††
Phosphate (mmol/L)	1.55 ± 0.33	1.59 ± 0.32	1.61 ± 0.40	1.64 ± 0.41	NS, NS, NS

Other causes of end-stage kidney disease included chronic interstitial nephritis, lithium nephropathy, vasculitis, postrenal disease, and unknown origin. Continuous variables as mean ± standard deviations or median (interquartile range); categorical variables as n (%). Independent-samples t-test, the Mann–Whitney U test, and/or Fisher’s exact test were performed for comparisons. Significance between the all-cause death and no event groups: *** *p* < 0.001, ** *p* < 0.01, * *p* < 0.05, NS, not significant. Significance between the cardiovascular death and no event groups: ### *p* < 0.001, ## *p* < 0.01, # *p* < 0.05, NS, not significant. Significance between the composite vascular event and no event groups: ††† *p* < 0.001, †† *p* < 0.01, † *p* < 0.05, NS, not significant.

## Data Availability

All data are fully available without restriction, in an anonymized format, upon request to the corresponding author.

## Supplementary Materials

The following supporting information can be downloaded at: https://www.mdpi.com/article/10.3390/biomedicines10040740/s1, supplementary method, Figure S1: Unadjusted hazard ratio (A) and adjusted hazard ratio (B) of all-cause death in PEA-based proteins using Cox regression analysis. Hazard ratios (HRs) were presented as a per SD increase in NPX value on proteomics. Multivariable Cox regression models were adjusted for age, gender, smoking status, cause of end-stage kidney disease, diabetes, previous myocardial infarction, previous unstable angina, previous cerebrovascular disease, previous treatment for peripheral artery disease, albumin, phosphate, and C-reactive protein, dialysis modality, dialysis treatment time per week, and dialysis vintage, Figure S2: Unadjusted hazard ratio (A) and adjusted hazard ratio (B) of cardiovascular death in PEA-based proteins using Cox regression analysis. Hazard ratios (HRs) were presented as a per SD increase in NPX value on proteomics. Multivariable Cox regression models were adjusted for age, gender, smoking status, cause of end-stage kidney disease, diabetes, previous myocardial infarction, previous unstable angina, previous cerebrovascular disease, previous treatment for peripheral artery disease, albumin, phosphate, and C-reactive protein, dialysis modality, dialysis treatment time per week, and dialysis vintage, Figure S3: Unadjusted hazard ratio (A) and adjusted hazard ratio (B) of composite vascular events in PEA-based proteins using Cox regression analysis. Hazard ratios (HRs) were presented as a per SD increase in NPX value on proteomics. Multivariable Cox regression models were adjusted for age, gender, smoking status, cause of end-stage kidney disease, diabetes, previous myocardial infarction, previous unstable angina, previous cerebrovascular disease, previous treatment for peripheral artery disease, albumin, phosphate, and C-reactive protein, dialysis modality, dialysis treatment time per week, and dialysis vintage, Figure S4: Correlation matrix between clinical variables and PEA-based proteomics biomarkers. The size and color of circles represent R values, Table S1: List of 96 protein biomarkers measured by proximity extension assay-based proteomic assay. Two proteins that failed quality control metrics were excluded from the analysis are indicated, Table S2. Outcomes (all-cause death, cardiovascular death, and composite vascular events) association in PEA-based proteins using Cox regression analysis and multiple testing by false discovery rate (FDR) adjusted *p*-value.
