# Supplementary material for: Novel Biomarkers Detected by Proteomics Predict Death and Cardiovascular Events in Hemodialysis Patients
Source: Biomedicines. 2022 Mar 22;10(4):740. doi: 10.3390/biomedicines10040740 (PMC9026983; doi:10.3390/biomedicines10040740)

Supplementary method

The detailed definition of the composite vascular event (CVE)

1. Acute myocardial infarction (AMI)

Acute myocardial infarction is diagnosed at least two of three criteria: (1) Cardiac ischemic chest pain is present (2) ECG findings meet the criteria for a definitive diagnosis during an event (New or presumed new significant ST-segment–T wave changes or new left bundle branch block or development of pathological Q waves in the ECG) and/or (3) Elevated level of cardiac enzymes were measured during an event.

2. Unstable angina

Unstable angina requiring hospitalization is defined as symptoms of myocardial ischemia at rest (chest pain or equivalent) or an accelerating pattern of angina with frequent episodes associated with progressively decreased exercise capacity with no evidence of acute AMI.

3. Ischemic stroke

Stroke is defined as an acute episode of neurological dysfunction caused by vascular injury lasting ≥24 hours.

4. Transient ischemic attack (TIA)

TIA is defined as sudden onset of neurological symptoms, presumed to be ischemic, resolving in less than 24 hours, clearly attributable to focal involvement of the central nervous system (or of the eye) with no signs of a corresponding recent cerebral infarction on brain imaging.

5. Hemorrhagic stroke

Hemorrhagic stroke is defined as an acute episode of focal neurological symptoms with the presence of cerebral hemorrhage in the appropriate territory on brain imaging (CT or MRI) caused by a nontraumatic intraparenchymal, intraventricular, or subarachnoid hemorrhage.

6. Peripheral artery disease (PAD) events

PAD events were defined as limb ischemia with major amputation or urgent peripheral revascularization for ischemia

Table S1. List of 96 protein biomarkers measured by proximity extension assay-based proteomic assay. Two proteins that failed quality control metrics were excluded from the analysis are indicated.

Abbreviation	Protein Analyte	UniProt ID	% Missing	Mean ± SD	Median (IQR)
AGRP	Agouti-related protein	O00253	0%	4.79 ± 0.85	4.8 (4.31 - 5.34)
AM	Adrenomedullin	P35318	0%	7.59 ± 0.41	7.68 (7.33 - 7.87)
Beta-NGF	Beta-nerve growth factor	P01138	0%	1.52 ± 0.51	1.62 (1.26 - 1.84)
BNP	Natriuretic peptides B	P16860	4.2%	4.25 ± 2.4	4.51 (2.7 - 5.95)
CA-125	Ovarian cancer-related tumor marker CA 125	Q8WXI7	1.2%	2.92 ± 1.44	2.78 (2.07 - 3.56)
CASP-8	Caspase-8	Q14790	0%	1.04 ± 0.7	1.04 (0.6 - 1.48)
CCL20	C-C motif chemokine 20	P78556	0%	6.05 ± 1.44	5.85 (5.14 - 6.61)
CCL3	C-C motif chemokine 3	P10147	0%	2.79 ± 0.75	2.81 (2.32 - 3.14)
CCL4	C-C motif chemokine 4	P13236	0%	7.11 ± 0.74	7.1 (6.7 - 7.51)
CD40	CD40L receptor	P25942	0%	10.71 ± 0.6	10.81 (10.38 - 11.12)
CD40L	CD40 ligand	P29965	0%	7.24 ± 1.08	7.42 (6.77 - 7.93)
CHI3LI	Chitinase-3-like protein 1	P36222	0%	7.6 ± 1.22	7.59 (6.78 - 8.34)
CSF-1	Macrophage colony-stimulating factor 1	P09603	0%	8.48 ± 0.36	8.56 (8.31 - 8.71)
CSTB	Cystatin-B	P04080	0%	7.56 ± 0.52	7.74 (7.22 - 7.92)
CTSD	Cathepsin D	P07339	0%	7.18 ± 0.52	7.23 (6.88 - 7.5)
CTSL1	Cathepsin L1	P07711	0%	5.55 ± 0.6	5.56 (5.2 - 5.86)
CX3CL1	Fractalkine	P78423	0%	6.65 ± 0.56	6.7 (6.36 - 7.03)
CXCL1	C-X-C motif chemokine 1	P09341	0%	8.54 ± 0.67	8.57 (8.17 - 8.96)
CXCL16	C-X-C motif chemokine 16	Q9H2A7	0%	4.21 ± 0.57	4.25 (3.84 - 4.55)

CXCL6	C-X-C motif chemokine 6	P80162	0%	7.39 ± 0.81	7.4 (6.88 - 7.9)
DKK-1	Dickkopf-related protein 1	O94907	0%	7.08 ± 0.64	7.16 (6.76 - 7.46)
ECP	Eosinophil cationic protein	P12724	0%	5.43 ± 0.66	5.58 (5.06 - 5.88)
EGF	Epidermal growth factor	P01133	0%	8.61 ± 1.07	8.77 (8.18 - 9.2)
EN-RAGE	Protein S100-A12	P80511	0%	3.5 ± 1.2	3.41 (2.71 - 4.2)
ESM-1	Endothelial cell-specific molecule 1	Q9NQ30	0%	3.05 ± 0.76	3.02 (2.58 - 3.46)
FABP4	Fatty acid-binding protein 4	P15090	0%	5.18 ± 0.62	5.37 (4.78 - 5.63)
FAS	Tumor necrosis factor receptor superfamily member 6	P25445	0%	9.8 ± 0.47	9.86 (9.53 - 10.07)
FGF-23	Fibroblast growth factor 23	Q9GZV9	0%	7.5 ± 1.49	7.78 (6.84 - 8.57)
FS	Follistatin	P19883	0%	5.04 ± 0.64	5.05 (4.63 - 5.46)
GAL	Galanin peptides	P22466	0%	5.74 ± 0.95	5.74 (5.21 - 6.34)
Gal-3	Galectin-3	P17931	0%	4.95 ± 0.56	5.03 (4.64 - 5.31)
GDF-15	Growth/differentiation factor 15	Q99988	0%	10.84 ± 0.82	10.84 (10.35 - 11.33)
GH	Growth hormone	P01241	0%	8.79 ± 1.59	9.17 (7.96 - 9.91)
HB-EGF	Heparin-binding EGF-like growth factor	Q99075	0%	8.02 ± 0.74	8.02 (7.61 - 8.45)
HGF	Hepatocyte growth factor	P14210	0%	7.63 ± 0.68	7.64 (7.26 - 7.96)
hK11	Kallikrein-11	Q9UBX7	0%	6.67 ± 0.54	6.71 (6.36 - 6.99)
HSP 27	Heat shock 27 kDa protein	P04792	0%	3.86 ± 0.69	3.86 (3.49 - 4.21)
IL-16	Interleukin-16	Q14005	0%	5.39 ± 0.55	5.49 (5.12 - 5.75)
IL-18	Interleukin-18	Q14116	0%	7.24 ± 0.74	7.27 (6.86 - 7.66)
IL-1ra	Interleukin-1 receptor antagonist protein	P18510	0%	4.79 ± 0.86	4.71 (4.25 - 5.18)
IL27-A	Interleukin-27 subunit alpha	Q8NEV9	0%	3.68 ± 0.6	3.69 (3.38 - 4.07)

IL-4	Interleukin-4	P05112	94%*	0.28 ± 0.1	0.23 (0.23 - 0.23)
IL-6	Interleukin-6	P05231	0%	5.44 ± 1.48	5.29 (4.47 - 6.13)
IL-6RA	Interleukin-6 receptor subunit alpha	P08887	0%	6.72 ± 0.53	6.73 (6.42 - 7.08)
IL-8	Interleukin-8	P10145	0%	6.93 ± 0.85	6.89 (6.42 - 7.3)
ITGB1BP2	Melusin	Q9UKP3	Missing*	--	--
KLK6	Kallikrein-6	Q92876	0%	6.58 ± 0.66	6.61 (6.2 - 6.96)
LEP	Leptin	P41159	0.6%	5.04 ± 2.07	5.03 (3.82 - 6.47)
LOX-1	Lectin-like oxidized LDL receptor 1	P78380	0%	6.77 ± 0.78	6.87 (6.34 - 7.26)
mAmP	Membrane-bound aminopeptidase P	O43895	22.7%	1.86 ± 1.23	1.88 (0.97 - 2.64)
MB	Myoglobin	P02144	0%	7.69 ± 0.67	7.8 (7.35 - 8.17)
MCP-1	Monocyte chemotactic protein 1	P13500	0%	10 ± 0.57	10.02 (9.69 - 10.34)
MMP-1	Matrix metalloproteinase-1	P03956	0%	2.8 ± 1.13	2.77 (2.17 - 3.46)
MMP-10	Matrix metalloproteinase-10	P09238	0%	8.63 ± 0.65	8.65 (8.23 - 9.02)
MMP-12	Matrix metalloproteinase-12	P39900	0%	8.04 ± 1.03	8.09 (7.42 - 8.65)
MMP-3	Matrix metalloproteinase-3	P08254	0%	2.86 ± 0.94	2.79 (2.29 - 3.39)
MMP-7	Matrix metalloproteinase-7	P09237	0%	8.92 ± 0.83	8.94 (8.46 - 9.44)
MPO	Myeloperoxidase	P05164	0.3%	4.59 ± 0.48	4.71 (4.37 - 4.88)
NEMO	NF-kappa-B essential modulator	Q9Y6K9	0%	2.27 ± 0.64	2.28 (1.91 - 2.64)
NT-pro-BNP	N-terminal pro-B-type natriuretic peptide	NA	0%	4.78 ± 0.4	4.84 (4.52 - 5.05)
OPG	Osteoprotegerin	O00300	0.3%	9.82 ± 0.7	9.89 (9.51 - 10.23)
PAPPA	Pappalysin-1	Q13219	0.9%	2.1 ± 0.85	2.05 (1.56 - 2.54)
PAR-1	Proteinase-activated receptor 1	P25116	0%	4.06 ± 0.49	4.12 (3.87 - 4.34)

PDGF subunit B	Platelet-derived growth factor subunit B	P01127	0%	8.19 ± 0.74	8.29 (7.86 - 8.63)
PECAM-1	Platelet endothelial cell adhesion molecule	P16284	0%	4.47 ± 0.54	4.55 (4.17 - 4.8)
PlGF	Placenta growth factor	P49763	0%	9.27 ± 0.58	9.35 (8.97 - 9.67)
PRL	Prolactin	P01236	0%	4.96 ± 1.03	4.85 (4.38 - 5.65)
PSGL-1	P-selectin glycoprotein ligand 1	Q14242	18.7%	0.57 ± 0.39	0.65 (0.25 - 0.83)
PTX3	Pentraxin-related protein	P26022	0%	2.52 ± 0.75	2.51 (2.08 - 2.97)
RAGE	Receptor for advanced glycosylation end products	Q15109	0%	6.41 ± 0.58	6.48 (6.09 - 6.8)
REN	Renin	P00797	0%	5.7 ± 1.27	5.72 (4.96 - 6.43)
RETN	Resistin	Q9HD89	0%	8.66 ± 0.69	8.69 (8.29 - 9.13)
SCF	Stem cell factor	P21583	0%	8.51 ± 0.5	8.61 (8.21 - 8.84)
SELE	E-selectin	P16581	0%	4.85 ± 0.9	4.81 (4.32 - 5.35)
SIRT2	SIR2-like protein 2	Q8IXJ6	0%	2.16 ± 0.83	2.17 (1.69 - 2.65)
SPON1	Spondin-1	Q9HCB6	0%	5.17 ± 0.48	5.24 (4.87 - 5.47)
SRC	Proto-oncogene tyrosine-protein kinase Src	P12931	0%	3.46 ± 0.7	3.44 (3.09 - 3.85)
ST2	ST2 protein	Q01638	0%	4.27 ± 1.04	4.16 (3.7 - 4.86)
TF	Tissue factor	P13726	0%	5.9 ± 0.53	5.97 (5.62 - 6.23)
TIE2	Angiopoietin-1 receptor	Q02763	0%	5.94 ± 0.44	6 (5.74 - 6.22)
TIM-1	T-cell immunoglobulin and mucin domain 1	Q96D42	0%	7.41 ± 1.72	7.35 (6.34 - 8.52)
TM	Thrombomodulin	P07204	0%	9.68 ± 0.36	9.74 (9.52 - 9.9)
TNF-R1	Tumor necrosis factor receptor 1	P19438	0%	12.67 ± 0.36	12.78 (12.57 - 12.9)
TNF-R2	Tumor necrosis factor receptor 2	P20333	0%	6.82 ± 0.38	6.95 (6.63 - 7.09)
TNFSF14	Tumor necrosis factor ligand superfamily member 14	O43557	0%	2.58 ± 0.74	2.58 (2.14 - 3.01)

tPA	Tissue-type plasminogen activator	P00750	0%	7.95 ± 0.94	7.97 (7.41 - 8.52)
TRAIL	TNF-related apoptosis-inducing ligand	P50591	0%	7.52 ± 0.54	7.61 (7.21 - 7.86)
TRAIL-R2	TNF-related apoptosis-inducing ligand receptor 2	O14763	0%	5.01 ± 0.69	5.03 (4.58 - 5.37)
TRANCE	TNF-related activation-induced cytokine	O14788	2.1%	3.14 ± 1.02	3.19 (2.58 - 3.76)
U-PAR	Urokinase plasminogen activator surface receptor	Q03405	0%	10.1 ± 0.35	10.18 (9.92 - 10.34)
VEGF-A	Vascular endothelial growth factor A	P15692	0%	11.69 ± 0.5	11.8 (11.39 - 12.02)
VEGF-D	Vascular endothelial growth factor D	O43915	0%	6.61 ± 0.53	6.68 (6.35 - 6.93)

* Exclude from the final data analysis because of the low quality of proteomics on IL-4 and missing data on ITGB1BP2.

Table S2. Outcomes (all-cause death, cardiovascular death, and composite vascular events) association in PEA-based proteins using Cox regression analysis and multiple testing by false discovery rate (FDR) adjusted *p*-value.

protein	All-cause death	CV death	CVEs
AGRP	0.1666	0.4737	0.3226
AM	0.7963	0.3805	0.8970
Beta.NGF	0.8570	0.3320	0.9656
BNP	**0.0057**	0.2811	**0.0002**
CA.125	0.1123	0.4365	0.2055
CASP.8	0.6833	0.3392	0.8067
CCL20	**0.0059**	0.1991	**0.0076**
CCL3	0.5459	0.9389	0.9599
CCL4	0.2802	0.7935	0.5009
CD40	0.6236	0.4140	0.9919
CD40.L	0.1191	0.5567	0.7116
CHI3L1	**0.0057**	0.2811	**0.0132**
CSF.1	0.9722	0.4720	0.6887
CSTB	0.9687	0.3320	0.8332
CTSD	0.1127	0.2811	**0.0132**
CTSL1	**0.0059**	0.3320	0.0555
CX3CL1	**0.0359**	0.2066	0.2663
CXCL1	0.8110	0.9610	0.0724
CXCL16	0.8643	0.3320	0.4052

CXCL6	0.9104	0.4140	0.4087
Dkk.1	0.4503	0.9604	0.4776
ECP	0.1123	0.3095	0.8930
EGF	**0.0395**	0.4140	0.8868
EN.RAGE	**0.0210**	0.2203	**0.0001**
ESM.1	**0.0193**	0.4380	0.3226
FABP4	0.1725	0.1991	0.5598
FAS	0.3999	0.4140	0.7001
FGF.23	0.5725	0.7475	0.6754
FS	0.0717	0.3095	0.2055
GAL	**0.0193**	0.3320	0.1733
Gal.3	0.3999	0.3320	0.3602
GDF.15	0.9961	0.7191	0.1250
GH	0.2869	0.3859	0.3602
HB.EGF	0.2414	0.4140	0.7386
HGF	**0.0059**	0.3320	**0.0217**
hK11	**0.0007**	0.2811	0.4871
HSP.27	0.8295	0.9272	0.0513
IL.16	0.2242	0.3320	0.7415
IL.18	0.1706	0.9460	0.3602
IL.1ra	0.1572	0.7873	0.2003
IL.6	**0.0007**	0.2811	**0.0001**

IL.6RA	0.7483	0.3320	0.8742
IL.8	**0.0057**	0.2811	**0.0118**
IL27.A	**0.0210**	0.9785	0.2663
KLK6	**0.0002**	0.1991	0.1577
LEP	0.6451	0.4140	0.5177
LOX.1	0.6530	0.4365	0.3298
mAmP	**0.0143**	0.2066	0.0698
MB	0.1123	0.3320	0.7976
MCP.1	0.2743	0.3095	0.9759
MMP.1	0.4251	0.3320	0.7801
MMP.10	0.2242	0.3320	0.9262
MMP.12	0.2797	0.5054	**0.0076**
MMP.3	0.3233	0.4892	0.8390
MMP.7	0.7019	0.3095	0.4087
MPO	0.7190	0.4443	0.6920
NEMO	0.8617	0.7351	0.3226
NT.pro.BNP	0.1725	0.3189	0.6910
OPG	**0.0221**	0.2811	**0.0112**
PAPPA	0.0773	0.9104	0.5598
PAR.1	0.2899	0.4967	0.3226
PDGF.subunit.B	0.3896	0.6059	0.5195
PECAM.1	0.2414	0.6214	0.2663

PlGF	0.8345	0.4365	0.9192
PRL	0.6975	0.6516	0.6076
PSGL.1	0.3848	0.3095	0.7300
PTX3	0.1725	0.9831	0.2055
RAGE	0.3797	0.3081	0.6552
REN	0.7476	0.3095	0.3761
RETN	0.9405	0.9951	0.4776
SCF	**0.0007**	**0.0157**	**0.0118**
SELE	0.5574	0.8846	0.2130
SIRT2	0.7546	0.7219	0.3298
SPON1	0.4703	0.7014	0.2887
SRC	0.2634	0.9036	0.7701
ST2	0.0876	0.3392	**0.0132**
t.PA	**0.0009**	0.0847	**0.0118**
TF	0.4677	0.5011	0.7960
TIE2	0.8336	0.9956	0.5708
TIM-1	**0.0274**	0.0847	**0.0076**
TM	0.5433	0.3320	0.9356
TNF.R1	0.2414	0.3095	0.5177
TNF.R2	0.2638	0.3095	0.5645
TNFSF14	0.9587	0.7891	0.2887
TRAIL	0.2482	0.7952	0.4875

TRAIL.R2	**0.0193**	0.4635	**0.0076**
TRANCE	0.0635	0.4419	0.0686
U.PAR	0.2899	0.3299	0.4668
VEGF.A	0.9353	0.3320	0.4087
VEGF.D	0.9150	0.5737	0.3321

Figure S1. Unadjusted hazard ratio (A) and adjusted hazard ratio (B) of **all-cause death** in PEA-based proteins using Cox regression analysis.

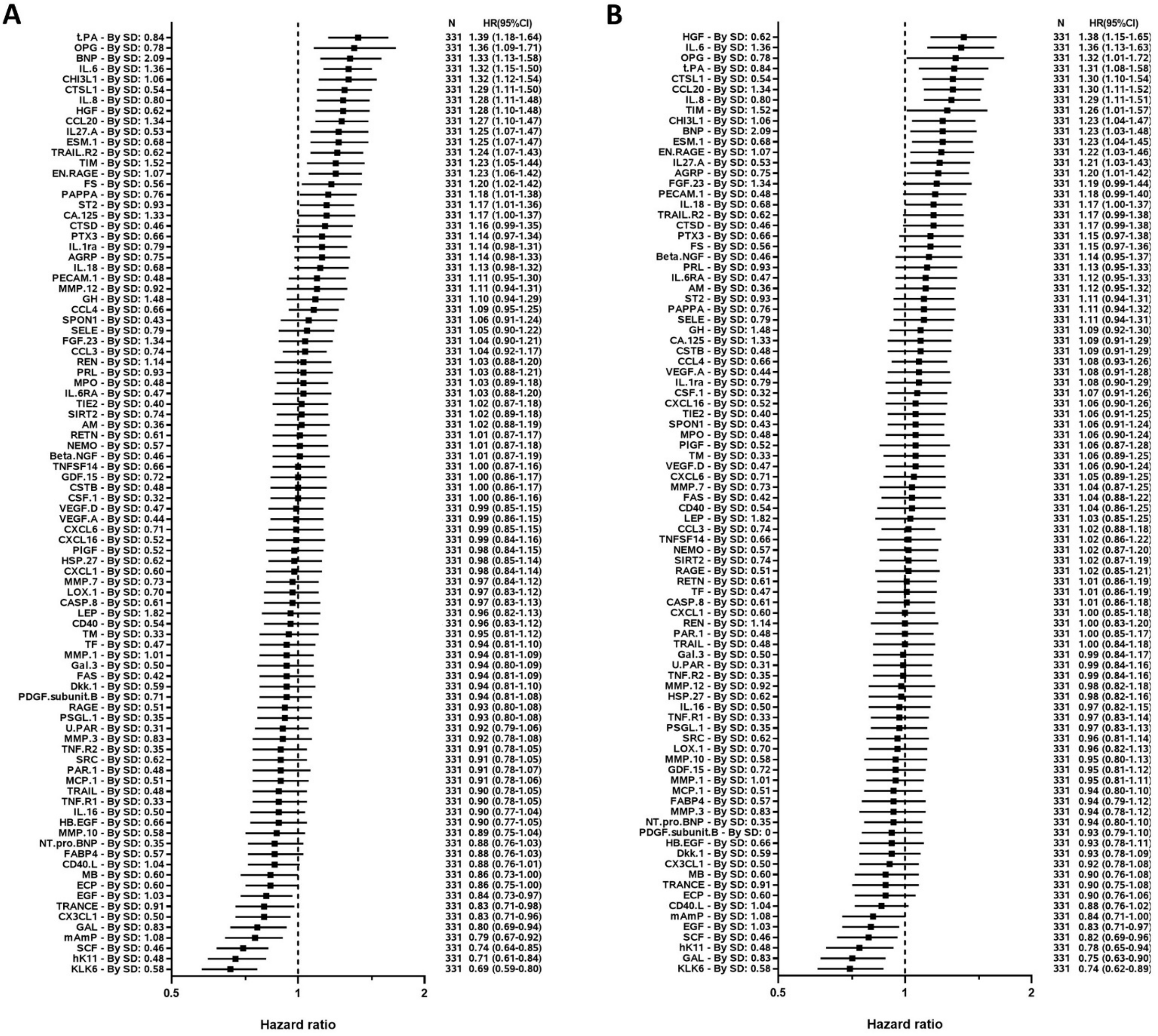

Hazard ratios (HRs) were presented as a per SD increase in NPX value on proteomics. Multivariable Cox regression models were adjusted for age, gender, smoking status, cause of end-stage kidney disease, diabetes, previous myocardial infarction, previous unstable angina, previous cerebrovascular disease, previous treatment for peripheral artery disease, albumin, phosphate, and C-reactive protein, dialysis modality, dialysis treatment time per week, and dialysis vintage.

Figure S2. Unadjusted hazard ratio (A) and adjusted hazard ratio (B) of **cardiovascular death** in PEA-based proteins using Cox regression analysis.

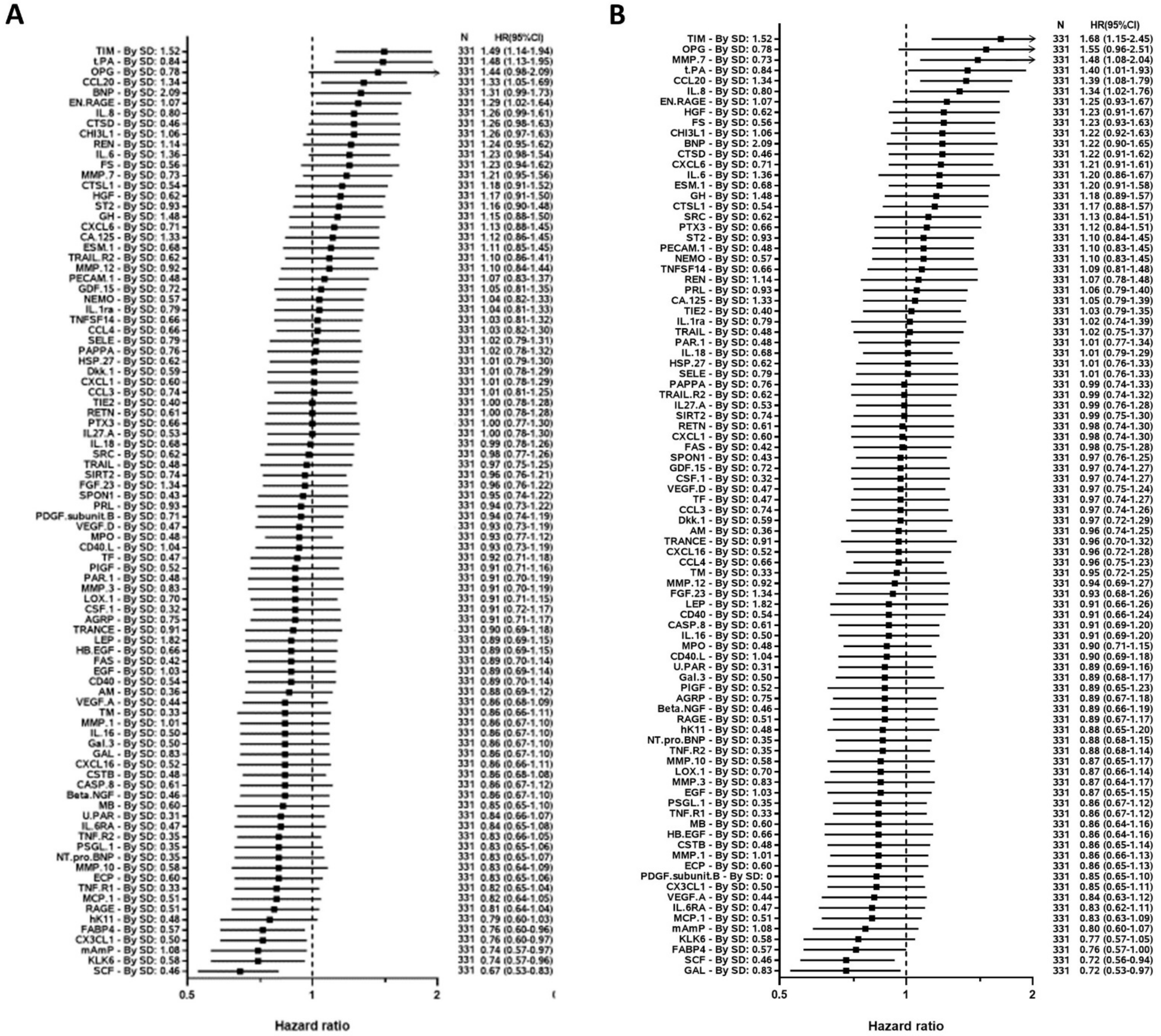

Hazard ratios (HRs) were presented as a per SD increase in NPX value on proteomics. Multivariable Cox regression models were adjusted for age, gender, smoking status, cause of end-stage kidney disease, diabetes, previous myocardial infarction, previous unstable angina, previous cerebrovascular disease, previous treatment for peripheral artery disease, albumin, phosphate, and C-reactive protein, dialysis modality, dialysis treatment time per week, and dialysis vintage.

Figure S3. Unadjusted hazard ratio (A) and adjusted hazard ratio (B) of **composite vascular events** in PEA-based proteins using Cox regression analysis.

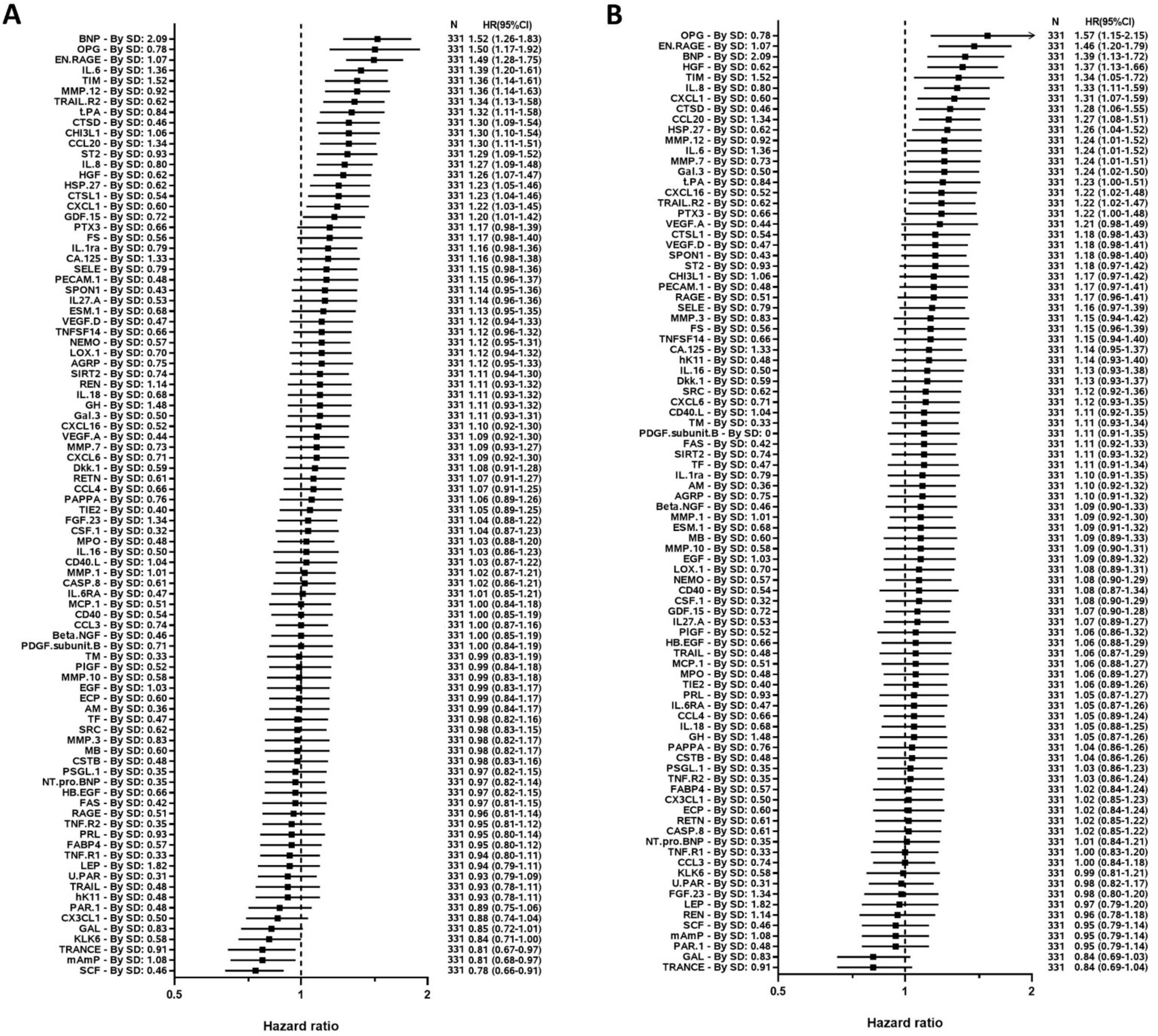

Hazard ratios (HRs) were presented as a per SD increase in NPX value on proteomics. Multivariable Cox regression models were adjusted for age, gender, smoking status, cause of end-stage kidney disease, diabetes, previous myocardial infarction, previous unstable angina, previous cerebrovascular disease, previous treatment for peripheral artery disease, albumin, phosphate, and C-reactive protein, dialysis modality, dialysis treatment time per week, and dialysis vintage.

Figure S4. Correlation matrix between clinical variables and PEA-based proteomics biomarkers. The contrast of circles represents the positive correlation (blue color) to the negative correlation (red color). The size of circles represents the R values of the relationship.

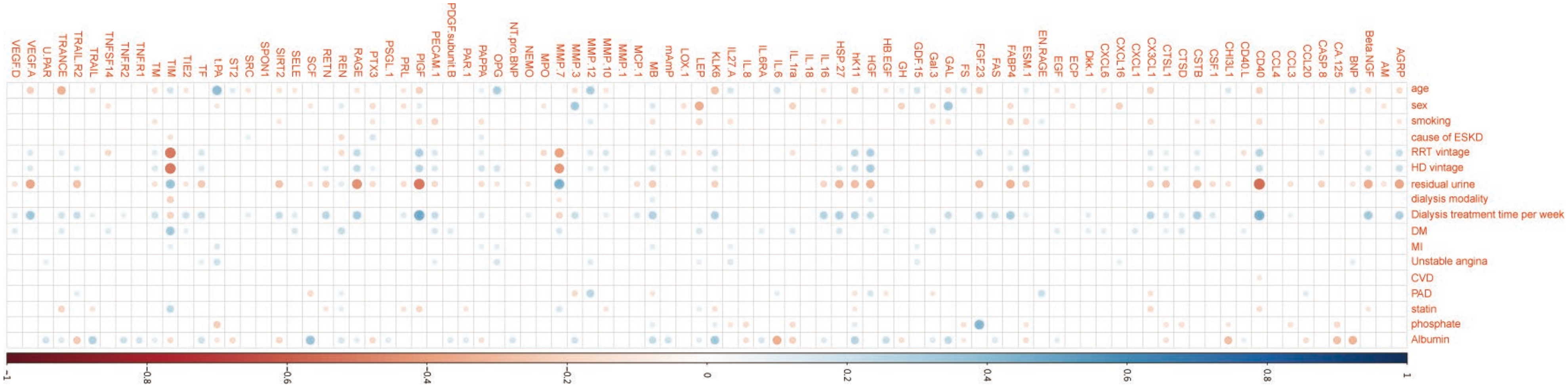

Supplement: Supplementary file 1 [file biomedicines-10-00740-s001.zip › All supplementary tables and figures (20220322).pdf]
